# Old Doctors or New?

**Published:** 1891-11-14

**Authors:** 


### THE DOCTOR'S ARMCHAIR.

OLD DOCTORS OR NEW?

Medical politicians are warming up. There is to be a contest after all for the vacant seats in the General Medical Council. The question seems to resolve itself into one of old friends and new; of tried and untried representatives. Dr. Glover is to have a walk over; Sir Walter Foster and Mr. Wheelhouse are to be, if possible, displaced. Indeed, if we mistake not, they have been asked to displace themselves, and that by one of the two gentlemen who are anxious to possess themselves of the seats to be vacated. Mr. George Brown, who, with Dr. Alderson, desires to become a member of the Medical Council, has, it seems, plainly intimated to Mr. Wheelhouse in a private letter that Sir Walter Foster and himself would be doing a graceful act if they would make way for Dr. Alderson and Mr. Brown. That certainly would be very nice from Mr. Brown's point of view, though whether it does not show a little more assurance than any medical man should permit himself to be the possessor of, we must leave Mr. Brown to decide.

Sir Walter Foster and Mr. Wheelhouse have, with Dr. Glover, represented the body of the profession on the Medical Council for five years, and the whole three now seek re-election. It is, therefore, a fit and proper occasion for taking the measure of their work. Moreover, it is right to ask them not only what they have done in the past, and how they have done it, but also what they intend to do in the future. We cannot compare the record of Messrs. Wheelhouse, Foster, and Glover with that of Messrs. Brown and Alderson, for the simple reason that the latter gentlemen have no record at all. We can, however, compare the programme sketched out by the two sets of candidates; and if it should appear that there is so little difference between the two programmes as to show them to be practically identical, then it seems to us that the three old candidates should be judged by their past performances, and accepted or rejected on the value of the work they have done, and the way in which they have done it.

It is proper to remember that Sir Walter Foster and his two colleagues were elected to represent the profession, and the whole of the profession, not a portion of it. And it is desirable that the elected representatives of the profession should represent the whole body of practitioners, and that for several reasons. Two or three of these may be here considered: and, first, it is to be borne in mind that the Medical Council, as a whole, is disposed to regard itself as a somewhat portentous organisation. Its business is, it considers, above all things, to be dignified; and to preserve, and conserve, and promote the dignity of the profession of which it deems itself the head. An organisation possessed with this idea is apt to be somewhat like the grand Llama. It feels itself too entirely precious for contact with the rough and common world, and it is afraid to move either backward or forward for fear that the glass shade under which it delights to live should be shattered to pieces.

A body thus constituted and inspired must be approached with exceeding skill and wisdom if it is to be moved to do any public work of importance at all. If the profession were to send into its presence two or three representatives possessed with the one idea that their business was to "prod up" the Council, as if it were an underfed and reluctant member of the "hee-haw" tribe, the result would be absolutely fatal to the possibility of any movement of any kind. But this "prodding" idea seems to be the one and only thought which occupies the heads of Messrs. Alderson and Brown. If those gentlemen were elected, they would only stand in the ratio of one to nine or ten. They would, in fact, be absolutely powerless: the chief effect of their prodding policy would undoubtedly be to make the Council coil itself up like an injured rodent of the prickly order, and to refuse to move at all.

A second thing to remember about such a preternaturally dignified body as the Medical Council is, that though it may be exceedingly slow to move of its own initiative, it is quite willing to go gently and cautiously forward if it can be shown that there is public honour and credit to be acquired by so doing. Here lies the opportunity of the elected members of the profession. They can take counsel with the great body of practitioners as to the most urgent needs of the profession: they can put those needs into the shape which they know by experience will be most acceptable to the non-elected members of the Council, and they can, in short, offer the dignified quadruped a tempting bunch of carrots instead of threatening him with a prod at the end of a stick.

Now the question for the great body of practitioners to ask themselves is this—Have their elected representatives, Sir Walter Foster, Mr. Wheelhouse, and Dr. Glover, shown themselves capable of so influencing their fellow members of the Medical Council as to get the whole body to move forward on safe and advantageous lines? There is but one answer to this question: they have done not only all that was possible, but more, we venture to think, than the most sanguine of practitioners expected of them. They have lengthened the medical curriculum, and improved preliminary education: they have safe-guarded the interests of the profession, and laboured to reconcile those interests with the public well-being: they have striven and are still striving to elevate the status of all medical practitioners both professionally and socially: they have acted with both energy and discretion; they have in fact been a good deal above the average of popular representatives. Tried by the most practical of tests they come out excellently well.

Why then should we think of dismissing our old friends and taking up with new? An established practitioner would not probably make a very great objection to a young fellow fresh from college putting up his brass-plate in the next street; but he would consider it extremely unkind if all or even a considerable number of his old patients were to go over to the new man at the very first opportunity. That kind of thing is not generally approved in the medical profession. Few of us have any very overpowering admiration for the Medical Council as a body: but we have ample reason to respect and even to admire those members of the Council whom we ourselves have elected. They have done many things well for us, and nothing ill: they are loyal in heart to the profession, and have the unspeakable advantage of having had five years' experience of their representative work. What wise man would desire to change them for new men with nothing but their brass-plates to recommend them? The profession must stand loyally by its old friends, and carry them to the top of the poll.

